# Activity Recognition in Individuals Walking With Assistive Devices: The Benefits of Device-Specific Models

**DOI:** 10.2196/rehab.7317

**Published:** 2017-08-10

**Authors:** Luca Lonini, Aakash Gupta, Susan Deems-Dluhy, Shenan Hoppe-Ludwig, Konrad Kording, Arun Jayaraman

**Affiliations:** ^1^ Shirley Ryan Ability Lab Max Näder Lab Chicago, IL United States; ^2^ Department of Physical Medicine and Rehabilitation Northwestern University Chicago, IL United States

**Keywords:** activities of daily living, machine learning, wearables, rehabilitation, orthotic devices

## Abstract

**Background:**

Wearable sensors gather data that machine-learning models can convert into an identification of physical activities, a clinically relevant outcome measure. However, when individuals with disabilities upgrade to a new walking assistive device, their gait patterns can change, which could affect the accuracy of activity recognition.

**Objective:**

The objective of this study was to assess whether we need to train an activity recognition model with labeled data from activities performed with the new assistive device, rather than data from the original device or from healthy individuals.

**Methods:**

Data were collected from 11 healthy controls as well as from 11 age-matched individuals with disabilities who used a standard stance control knee-ankle-foot orthosis (KAFO), and then a computer-controlled adaptive KAFO (Ottobock C-Brace). All subjects performed a structured set of functional activities while wearing an accelerometer on their waist, and random forest classifiers were used as activity classification models. We examined both global models, which are trained on other subjects (healthy or disabled individuals), and personal models, which are trained and tested on the same subject.

**Results:**

Median accuracies of global and personal models trained with data from the new KAFO were significantly higher (61% and 76%, respectively) than those of models that use data from the original KAFO (55% and 66%, respectively) (Wilcoxon signed-rank test, *P*=.006 and *P*=.01). These models also massively outperformed a global model trained on healthy subjects, which only achieved a median accuracy of 53%. Device-specific models conferred a major advantage for activity recognition.

**Conclusions:**

Our results suggest that when patients use a new assistive device, labeled data from activities performed with the specific device are needed for maximal precision activity recognition. Personal device-specific models yield the highest accuracy in such scenarios, whereas models trained on healthy individuals perform poorly and should not be used in patient populations.

## Introduction

Activity recognition (AR) has become an active area of research in the past decade, largely driven by the availability of low-cost wearable sensors and general purpose machine learning algorithms [1,2]. A promise of such systems is to unobtrusively track and quantify daily physical activities or other physiological parameters and ultimately provide personalized recommendations to prevent health problems or tailor exercise or rehabilitation programs.

Rehabilitation is an area of health care that can largely benefit from AR [3]. By monitoring functional activities of individuals with disabilities, clinicians and researchers can rely on quantitative data to evaluate the effectiveness of a treatment or an assistive device and optimize them to improve patient outcomes. This need is fueled by the rapid development of novel prostheses, orthoses, and wearable robots that can recognize the user intentions or the environment properties and adapt the device’s mechanical properties accordingly [4,5]. In order to justify reimbursement of such devices from health insurance companies, clinical studies need to provide quantitative evidence that this technology significantly improves a patient’s quality of life, compared with conventional assistive devices. Therefore, AR systems can overcome the limitations of current clinical tests in collecting such data.

The majority of wearable- and mobile phone–based AR studies have been conducted using healthy individuals, whereas relatively fewer studies are focused on people with disabilities [6], such as those resulting from stroke [7-9] or Parkinson disease [10,11]. Some of these studies showed that a model trained on young healthy individuals will yield poor performance when used with a different population [9,11-13], including those who need an assistive device for walking [14]. These differences arise due to the fact that movements are unique to individuals, and movements in people with a disability are different from that of able-bodied individuals [15]. As a result, AR systems are still of limited use in health care applications [16].

Furthermore, gait patterns of individuals with disabilities can change significantly from that of healthy individuals, and additional variability can arise when disabled individuals who walk with an assistive device switch to a new device. The source of such variability can be due to differences in the mechanical design or in the way the new device is controlled, which often requires the person to learn new movement strategies [4]. These differences could affect the reliability of an AR model and should be considered when deploying an AR system for clinical purposes.

In general, an AR model can be user specific (*personal* model) or it can be trained on data from other individuals to predict the activities of a new individual (*population* or *global* model). Global models are arguably easier to deploy, as they do not require labeled data from every new user; in addition, they can be trained on a larger dataset, as data from many users are aggregated to train the model. However, their lack of specificity can affect accuracy [17], due to the variability that exists between individuals. Personal models, in contrast, are trained on data from each new subject, with the advantage of being highly specific. However, collecting labeled data from each new subject is expensive. Thus, it is important to understand under which conditions a model will perform well.

Studies comparing personal with global models showed mixed results [2], with some emphasizing the need of using personal models [18] whereas others reporting that global models can be flexible enough to generalize to new users [19]. Few approaches attempted to enhance the performance of global models with unlabeled [20] or labeled [21] data from the new user or by combining activity models from other users with similar characteristics [22]. However, it is unclear how all these results will apply to patient populations, specifically those using different assistive devices.

Here we focus on identifying physical activities using a waist-worn accelerometer in people walking with a leg orthosis, namely a knee-ankle-foot orthosis (KAFO). A KAFO is normally used by individuals who suffered a traumatic or neurological injury, as well as a neuromuscular disease causing weakness or partial paralysis of one or both legs [23]. In our scenario, the persons with disabilities are testing a novel computer-controlled hydraulic KAFO (Ottobock C-Brace) that substitutes their control KAFO. We ask whether an AR model has to be trained with labeled data from the person performing physical activities with the C-Brace or whether data obtained from the control device or from other individuals will suffice. We analyze how the specificity of the training data affects the performance of the model as we move from a model trained with data from other subjects (global model) to one specific for each subject and brace (personal device-specific model).

## Methods

### Study Design

After being consented, 11 individuals with disabilities (3F, mean age 56.4 [SD 12.9] years) and 11 age-matched, able-bodied individuals (5F, mean age 49.2 [SD 19.4] years) participated in this study. Northwestern University’s Institutional Review Board approved the experimental procedures for the study, which took place at the Rehabilitation Institute of Chicago. For the sake of convenience, in the following, we will also refer to our pool of participants with disabilities as “patients.”

All patients required the use of a unilateral KAFO to ambulate due to either a neurological or traumatic injury or a neuromuscular disease causing muscular weakness in one leg (see Table 1). The recruited participants were part of a larger study that investigated whether a microprocessor-controlled KAFO (C-Brace) helps differently abled persons to better perform functional everyday activities and to have a more active lifestyle. All patient participants were able to transfer to sitting and standing and walk independently or with the supervision of a caregiver. Out of the 11 patients, 2 were not able to safely manage going up and down a flight of stairs and did not require stair climbing in their homes. The speed of walking and daily distance of walking varied within the patient population.

**Table 1:** Demographics of participants with disabilities.

Subj #	Gender	Age, in years	Diagnosis	Control assistive device
1	M	64	Poliomyelitis	Freewalk - Ottobock
2	F	59	Spinal cord injury	SPL2 - Fillauer
3	M	40	Poliomyelitis	E-MAG - Ottobock
4	M	64	Poliomyelitis	E-MAG - Ottobock
5	F	41	Poliomyelitis	E-MAG - Ottobock
6	M	35	Spinal cord injury	E-MAG - Ottobock
7	M	72	Poliomyelitis	E-MAG - Ottobock
8	M	68	West Nile meningitis	E-MAG - Ottobock
9	F	44	Peripheral neuropathy	Becker Stride - Becker
10	M	65	Poliomyelitis	E-MAG - Ottobock
11	M	68	Spinal cord injury	E-MAG -Ottobock

Each patient was fitted and effectively trained at using a passive stance-control KAFO as their control device and a microprocessor-controlled hydraulic KAFO as their novel device, namely the C-Brace (Ottobock, Duderstadt, Germany). Each device was used by the participants at home and in the community. Unlike traditional KAFOs, the C-Brace embeds a computer-controlled hydraulic unit that dynamically changes the impedance of the knee joint by using sensors in the knee and ankle joint that infer the slope of the ground surface and the user intent [4]. This stance and swing impedance feature assists the user in performing stand-to-sit movements as well as walking on a variety of surfaces and descending stairs.

All subjects wore a triaxial accelerometer (Actigraph wGT3X-BT; Actigraph LLC, Pensacola, FL) that recorded data at a sampling frequency of 30 Hz and was strapped around their waist on the right side with a belt. We aimed at detecting the following 5 functional activities: sitting, stair climbing and descent, standing, and walking. All subjects performed a scripted sequence containing the 5 activities, over 3 different sessions, which took place on separate days. Here, we define a single repetition of the sequence as a “session.” The total time of the recordings for each patient lasted an average of 35 minutes.

During each session, subjects were asked to sit comfortably while talking, gesturing, or checking their phone. They were then asked to stand while washing their hands or pouring and drinking water. Participants then walked at a self-selected, comfortable pace, and finally ascended and descended at least one flight of stairs at a self-selected pace. Each activity was performed for at least 30 seconds to ensure that enough data were collected. For safety purposes, all individuals with disabilities were supervised by a physical therapist.

Healthy subjects performed the scripted activities 3 times during 1 session. Patients performed the scripted activities during clinical training. For this data analysis, 3 sessions using the control assistive device and 3 using the novel assistive device were used. The sessions took place over a 3-week period on average. Due to comfort and safety issues related to their disability when using the new device, 2 patients could not ascend or descend stairs. A researcher observed the sessions and recorded the length of the activities for subsequent data labeling. Furthermore, all patients were administered the Orthotics Prosthetics Users Survey self-report questionnaire for lower extremity functional status (OPUS-LEFS) at the end of the study, to rate their level of comfort in using each KAFO. On average, all participants rated both the control and the novel device equally comfortable.

### Activity Recognition

**Figure 1:**
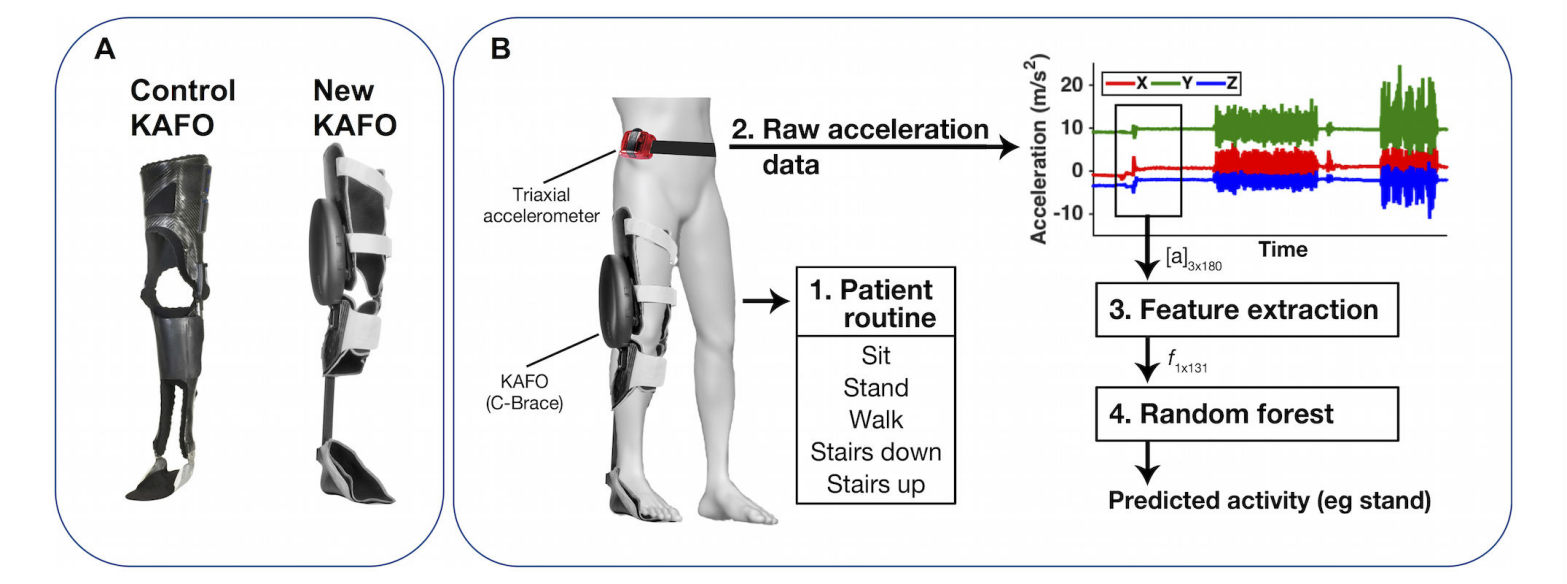
A. The two types of assistive devices (knee-ankle-foot orthosis, KAFO) used in the study. Patients performed activities with their control KAFO (passive stance-control orthosis) and then with the novel KAFO (Ottobock computer-controlled C-Brace). B. Experimental setup, data processing, and activity recognition steps (adapted with permission from [14]). A patient performed a set of activities while wearing a KAFO and a triaxial accelerometer. Windows of 6 seconds were extracted from the raw acceleration data (sampled at 30 Hz) yielding a matrix [a] of size 3×180. A set of 131 features were computed on each window, and the resulting vector f was inputted to a random forest classifier, which predicts the performed activity.

Accelerometer data were downloaded on a personal computer using the Actigraph ActiLife software (Actigraph LLC, Pensacola, FL). Data windows of 6 seconds with 75% overlap were extracted from the raw acceleration data and a set of 131 features (Table 2) were computed on each window. Both time and frequency domain features were used, as in previous studies [24]. The window length was selected based on previous AR studies that aimed at recognizing functional daily activities, such as walking or stair climbing [2,25] using wearable sensors. A random forest classifier [26] was used to predict the activity given a vector of features calculated on each window (Figure 1).

We selected random forest as it does not suffer from overfitting, performs well in activity recognition problems [27], and it has fewer hyper-parameters to optimize as compared with other classification models (eg, support vector machines). The number of trees was optimized to maximize the balanced accuracy (see the section “Performance Metric”), which resulted in 10 trees for the *Healthy* model and 50 trees for all the other models.

**Table 2:** List of features computed on the accelerometer data used for activity classification.

Description	Number of features
Mean, range, interquartile range (*x*, *y*, *z*)	9
Moments: standard deviation, skew, kurtosis (*x*, *y*, *z*)	9
Histogram: bin counts of −2 to 1 *z*-scores (*x*, *y*, *z*)	12
Derivative of moments: mean, standard deviation, skew, kurtosis (*x*, *y*, *z*)	12
Mean of the squared norm	1
Sum of axial standard deviations	1
Pearson correlation coefficient, *r* (*xy*), *r* (*xz*), *r* (*yz*)	3
Mean cross products (raw and normalized), *xy*, *xz*, *yz*	6
Absolute mean of cross products (raw and normalized)	6
Power spectra: mean, standard deviation, skew, kurtosis (*x*, *y*, *z*)	12
Mean power in 0.5 Hz bins between 0 and 10 Hz (*x*, *y*, *z*)	60

**Figure 2:**
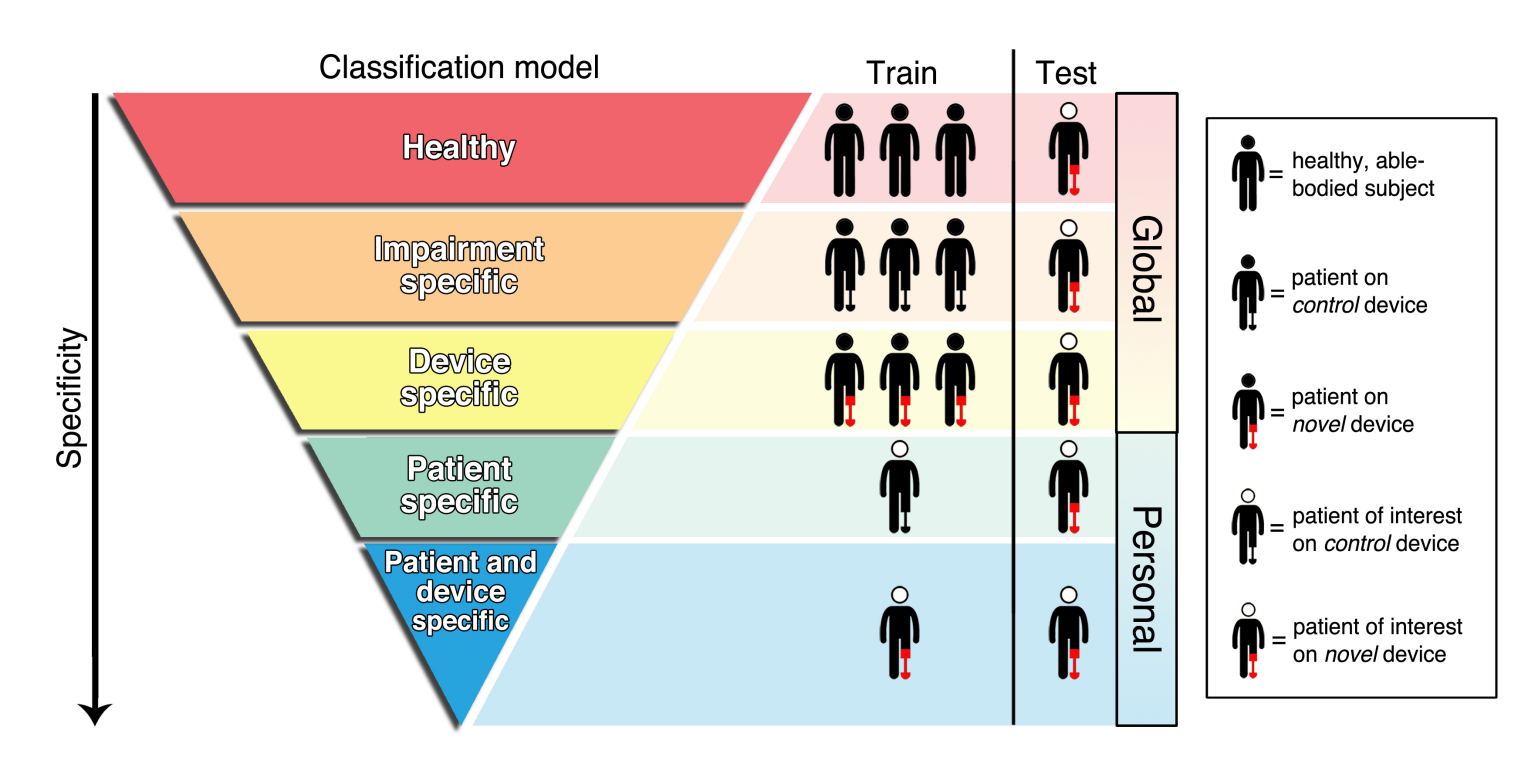
Diagram depicting increasing specificity of classification models in terms of what groups of individuals (able-bodied or individuals with disabilities/patients) they are trained on. Patients are depicted using their control (black) or novel (red) assistive device. Each classification model is used to predict activities for the patient of interest (Test), walking with the novel assistive device. The top 3 layers of the pyramid contain global models, which are trained on individuals other than the one used to test the model. The 2 bottom layers of the pyramid contain personal models, which are trained and tested with data from the same individual.

We trained 5 classification models (Figure 2) to compare how the training data affected classification accuracy when predicting each patient’s activities performed with the novel assistive device. Classification models are divided into 2 categories: *global models*, which are trained on data from subjects other than the one being tested, and *personal models*, which are trained and tested using data from the same subject.

#### Global Models

*Healthy model*: a classifier is trained on data collected from the healthy subjects (~9000 data points) and evaluated on each patient while using the novel device.

*Impairment-specific model*: a classifier is trained on data from other patients while using their control device (~16,000 data points), and evaluated on the patient of interest while using the novel device.

*Device-specific model*: a classifier is trained on data from other patients while using the novel device, and evaluated on the patient of interest while using the novel device.

#### Personal Models

*Patient-specific model*: each personal classifier is trained on a patient’s own control device data and evaluated on their novel device data (~1500 data points).

*Patient- and device-specific model*: each personal classifier is trained on a patient’s own novel device data and evaluated on their data using a leave-one-session-out cross-validation (~1000 data points).

### Performance Metric

As stair-climbing data are largely underrepresented, there is a significant class imbalance in the dataset. Because of that, we used the balanced accuracy (mean recall) as the metric to assess classifier performance, such that the error in each class receives equal weight. In scenarios with class imbalance, it is important to use an unbiased performance metric, such as the balanced accuracy or balanced error rate, to prevent drawing erroneous conclusions about the performance of the AR model [28].

*Balanced accuracy* = 1/
*C* Σ
_i=1:C_ (
*TP*_i_ /
*n*_i_)

where *C* is the number of activities (5 in our case), *TP*_i_ the number of true positives for activity *i*, and *n*_i_ the number of data points for activity *i*. Put simply, the balanced accuracy averages the prediction accuracy for each activity and, consequently, is not affected by the presence of more data for some activities. Class imbalance stems from the fact that patients using a KAFO can have difficulty ascending and descending stairs. However, these 2 activities are still performed by patients to some extent and, thus, are important in the assessment of a clinical AR system.

To compare performances across models we performed 4 Wilcoxon-signed rank tests to account for the non-normality of one of the distributions (Shapiro-Wilk test). These 4 tests were performed sequentially, such that each classification model was compared with the next more specific model, with alpha=.05.

### Training Data Size in Global Models

Whereas personal models are trained on data from a single subject, global models are trained on data from multiple subjects. As the number of subjects in the training dataset increases, the amount of training data increases, and the classification error of a global model will likely decrease. Therefore, we evaluated the balanced accuracy of both global models (healthy and impairment-specific) as a function of the number of training subjects. For each selected number of subjects, we ran 1000 training iterations, where in each iteration we randomly picked subjects to train on and one patient’s novel device data to test on. We chose 1000 iterations to account for a sufficient number of combinations of training and test subjects and for minor fluctuations in performance of the random forest. The largest number of training subjects for the impairment-specific model is 1 minus the total number of patients, as 1 patient is always set aside for testing. For each set of models trained on a selected number of subjects, we inferred the mean and 95% confidence interval of the median balanced accuracy by bootstrap using 1000 repetitions.

## Results

We compared the performance of global and personal classifiers trained with either data from patients who used their control KAFO assistive device or the novel C-Brace assistive device. A global model trained on healthy subjects was included in the comparison, representing the least specific classification model. Models were compared based on their balanced accuracy. Global models were then compared in terms of the amount of training data (number of subjects) used to reach a certain level of accuracy.

### Classifier Specificity

**Figure 3:**
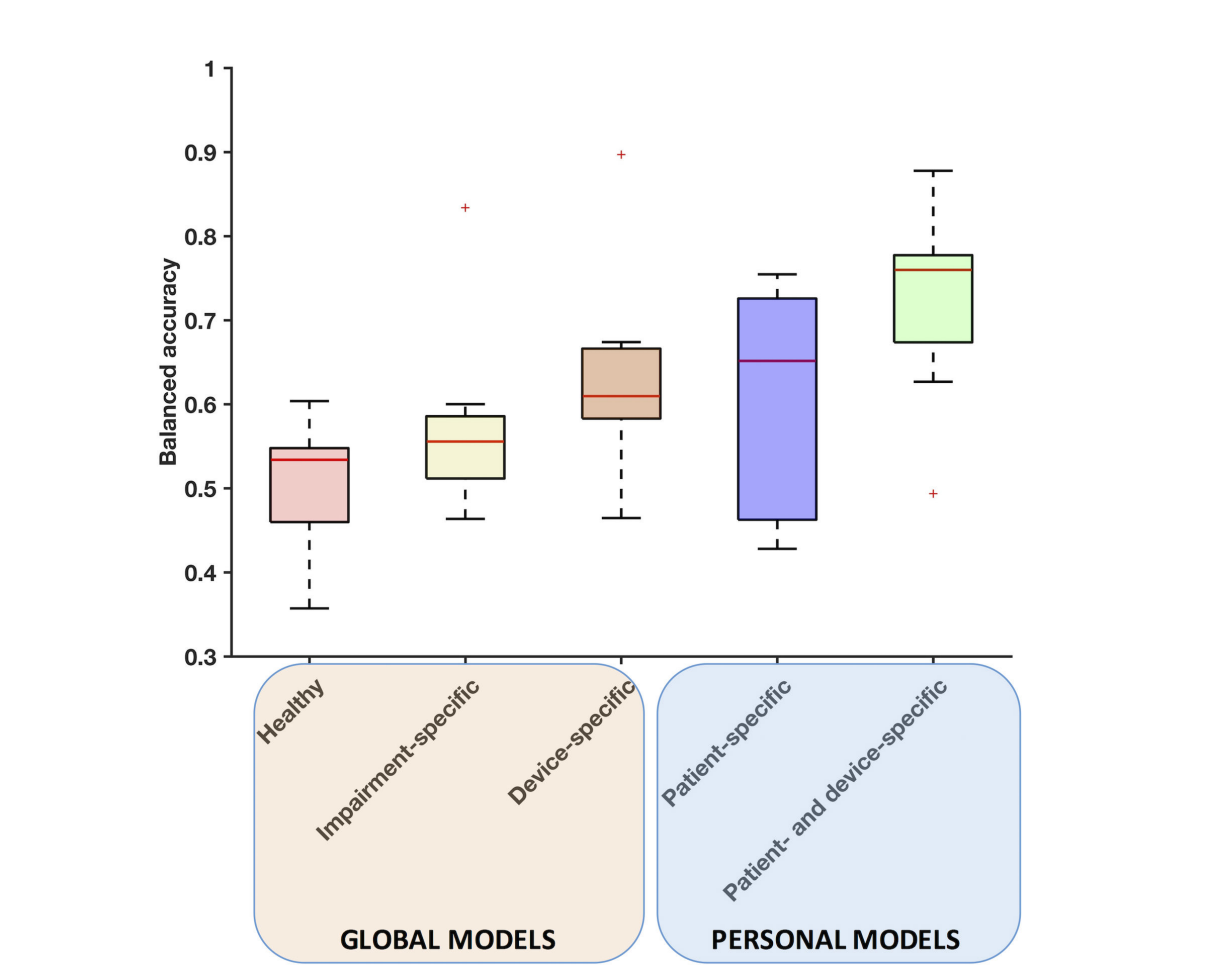
The distribution of balanced accuracies for the 5 models. Each model is tested on each patient using the novel assistive device (C-Brace). Boxes represent the interquartile range (IQR), red lines are medians, and whiskers show 1.5 IQR. Red crosses are outliers.

To understand whether training data from the novel assistive device will improve performance of a global model, we compared the classification accuracy across the 3 global models (Figure 3). A classifier trained with only healthy subjects’ data yielded the lowest balanced accuracy, with a median of 53%, for predicting the activities of a patient using the novel assistive device. A global model trained on patients using their control KAFO (impairment-specific) only performed marginally better (*P*=.03) than the healthy model, with a median balanced accuracy of 55%. In contrast, a global model trained using data from the novel device (device-specific) boosted the balanced accuracy significantly over the former 2 models (*P*=.006), reaching a value of 61%. Thus, data from activities performed with the specific assistive device used should be collected to achieve the highest accuracy with an AR system.

We then examined whether training data from the novel device affected the accuracy of personal models. The patient-specific model, which is a personal model trained with a patient’s control device data and tested on the patient’s own novel device data, yielded a median balanced accuracy of 66%. However, the performance of this model varied drastically across patients (interquartile range, IQR=[47%-72%]), and overall there was no statistically significant improvement over the global device-specific model (*P*=.29). Model accuracy did not correlate with how comfortable patients felt using the novel device, as measured by the OPUS-LEFS questionnaire (*r*=0.14, *P*=.69), indicating that the variable performance of the model is not related to the perceived comfort in using the device. This suggests that a personal model might overfit to the data from the control assistive device, and therefore, it does not confer an advantage over a global device-specific model.

Conversely, a personal model trained with the novel device data (patient- and device-specific) yielded the highest median balanced accuracy (76%), providing a significant advantage over all the previous models (*P*=.01). Of notice, this model was trained with the least amount of data (~1000 samples) across all models, which is about one-third less data than the patient-specific model. Therefore, regardless of whether a model is global or personal, the resulting classifier will perform significantly better if trained on data from the specific assistive device used by the patient.

**Figure 4:**
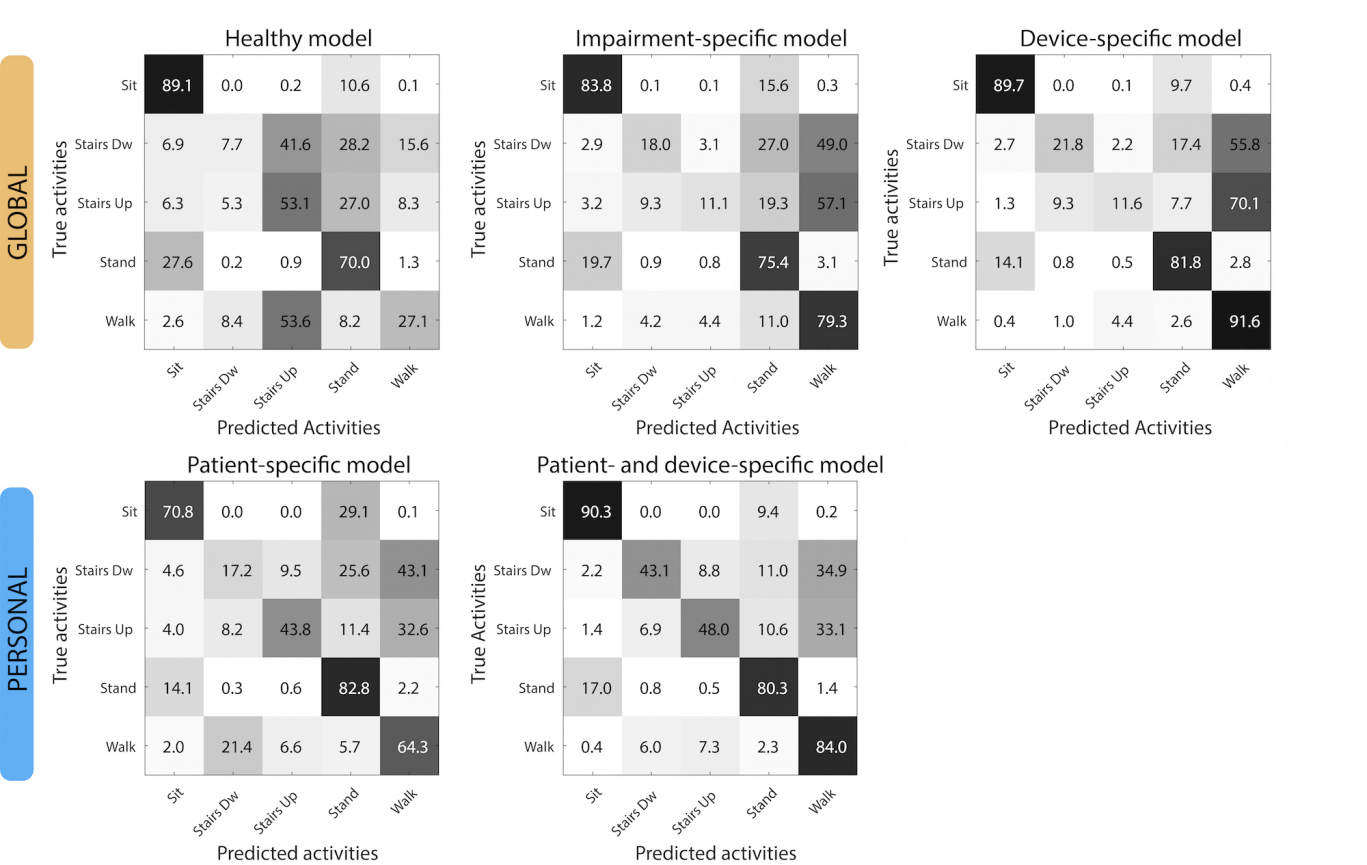
Confusion matrices for the 5 classification models, grouped by global and personal models. Numbers represent percentage of instances in that class.

As the results on the balanced accuracy do not reveal which activities are misclassified by each model, we analyzed the accuracy per class (recall) across the 5 activities for all models (Figure 4). The recall for sedentary/stationary activities (sitting and standing) was overall high for all models (>70%) and did not change dramatically across them. This is not surprising, as features used by each model to identify these activities are not expected to depend on the patient population, nor on the assistive device used.

The global healthy model had the lowest recall for predicting walking (27.13%, 1337/4928), which was mostly misclassified as climbing upstairs (Figure 4, top-left). Interestingly, recall for climbing upstairs had the highest value (53.1%, 331/623) compared with all other models, suggesting that features describing climbing upstairs might be similar between healthy subjects and patients walking with the novel device. In contrast, recall for climbing downstairs was quite low (7.7%, 45/582). This is surprising in that the C-Brace allows the knee to bend and support the user in a step-over-step stair descent similar to the pattern used by the healthy subjects. Thus, models trained on able-bodied displayed poor performance for capturing dynamic activities in patients.

On the other hand, recall for walking was significantly higher (79.26%, 3906/4928 and 91.61%, 4514/4928, respectively) in the impairment-specific and device-specific models (Figure 4, top-center and top-right), although both models misclassified most of the stair-climbing data (≤21.8%, 127/582) as walking. Consequently, global models trained on patients generalized well to walking data but were still poor at capturing stairs ascend and descend activities.

Patient-specific models performed in between the global-healthy model and the global-patients’ models, with a recall of 64.33% (3170/4928) for walking and of 43.8% (273/623) for stair climbing up. Recall for stair climbing down was still low (17.2%, 100/582). Recognition of both stair-ascend and descend activities only improved with the patient- and device-specific model (43.1%, 83.7/194 and 48.0%, 99.7/207.7), although the recall was well below that for walking or other activities. Therefore, the main gain achieved by personal models trained with the new device data was on the recognition of stair-climbing activities.

### Effect of Number of Subjects on Global Models

**Figure 5:**
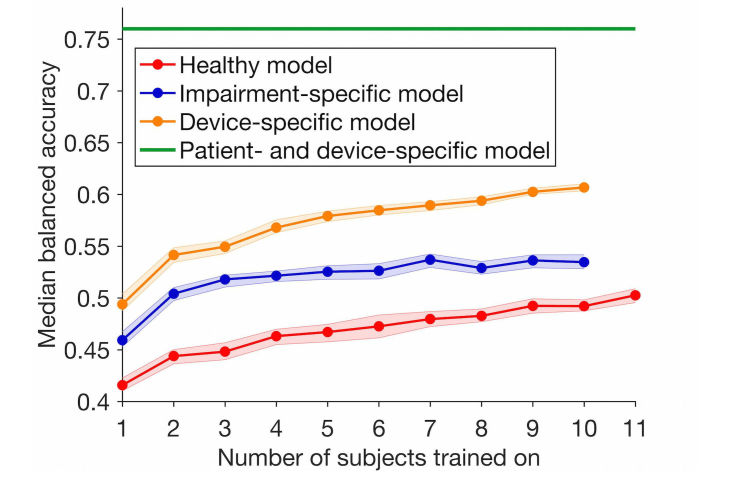
Effect of number of subjects used to train each global model on the median accuracy for healthy (red), impairment-specific (blue), and device-specific (orange) global models. The maximum number of subjects for patient models is 10, as 1 patient is left out for testing (leave-one-subject-out cross-validation). Shaded areas represent the 95% confidence intervals on the medians obtained by bootstrap. The green line represents the median accuracy of the patient- and device-specific models (personal model).

As global models are trained with data from multiple subjects, we evaluated how many subjects are required to achieve a desired level of performance for each global model. As expected, the median balanced accuracy increased with the number of subjects for all 3 global models (Figure 5). The median accuracy of the impairment-specific models seemed to plateau already with 11 subjects. However, trends for the Healthy and device-specific models suggest a further increase in accuracy if additional subjects are added. Nevertheless, the device-specific model showed a net advantage over the healthy and impairment-specific model, as a model trained on 1 patient performed as well as a model trained on 11 healthy individuals. Therefore, device-specific global models require significantly less data from patients to achieve the same performance, as compared to the other global models.

## Discussion

### Principal Findings

We asked whether AR models for individuals walking with an assistive device (KAFO) require training data from the new KAFO (C-Brace) or whether data from their control KAFO will suffice. We found that both global and personal models performed significantly better when trained with data from the novel KAFO used by the subjects to perform the functional activities. Therefore, an AR system has to be trained with data specific to the assistive device used to maximize classification accuracy.

We examined both global and personal models. Although global models were trained with about 16 times more samples than personal models, a personal model trained on the novel KAFO data (patient- and device-specific) largely outperformed all global models. Interestingly, this was not the case for a personal model trained on the control KAFO data (patient-specific), as the accuracy of this model was highly variable across subjects and overall not better than that of a global device-specific model. Therefore, in this scenario, a personal model might only help if trained with data from the specific assistive device used.

On the other hand, global models are arguably easier to deploy, as they do not require collecting data on each and every new patient [14]. Interestingly, in our scenario, personal device-specific models surpassed global models only for identifying stair-climbing activities, while being equally accurate at detecting walking. This suggests that when stair climbing is not a predominant daily activity that needs to be identified for a patient, a global device-specific model will equal the mean accuracy of a personal model.

Although the performance of the global-healthy model increased with the number of training subjects, this model was outperformed by global models trained on patients using the novel KAFO (device-specific). One reason is that gait patterns in individuals with disabilities can be markedly different from those of able-bodied subjects [15], and the algorithms could use different sensor features to identify activities in different populations [9,29]. Indeed, former studies found that activity recognition models trained on a population of young able-bodied individuals generalize poorly to patient populations, such as the elderly or patients of stroke or Parkinson’s disease [9,11-13]. Our findings are in line with these results and show that additional variability can be introduced by the use of different KAFOs. Therefore, a model trained on able-bodied individuals will likely be inaccurate when applied to a population that uses a KAFO to walk.

### Limitations

There were certain limitations to our study that we need to acknowledge. We only had a sample of 11 individuals with disabilities (patients) for training the global models; adding more subjects could increase the performance of these models, and should be explored in future studies. It has to be noted though that the accuracy of global models was dramatically lower than that of personal device-specific models. As reported by some prior studies, global models might not reach the performance of personal models even when a large number of subjects are used [18]. On the other hand, a global device-specific model equaled the performance of a patient-specific personal model, which suggests that personal models may suffer from overfitting to the specific assistive device used, and therefore, not generalize well across different assistive devices.

We asked our subjects to perform a structured set of activities in a lab setting and under the supervision of a clinician. Although specific instructions on how to perform activities were not provided (eg, washing hands or checking the phone), this scenario is still different from a natural environment. Previous studies showed that the accuracy of AR can drop significantly when the data collection is performed outside of a lab-controlled condition [30], and therefore, these findings should be validated outside of the lab. However, collecting labeled data in naturalistic environments remains a challenge, particularly with patient populations.

We compared performance of global models to that of personal models. However, one can also use intermediate approaches, where both data from other subjects and personal data are combined to train a new model. For example, activity-specific personal models from other subjects can be combined to fit a small dataset of labeled data from the target subject (semipopulation models) [31]. Such an approach can be guided by individual characteristics of the target individual, such as height and weight [32]. Transfer learning methods can also be employed: here, features learned in one domain, where data are abundant (eg, healthy or patient), are modified to fit the data in the target domain (eg, new patient or new assistive device), where labeled data are scarce or expensive to collect [28,33]. While we are investigating the application of these methods, further validation in a larger pool of subjects is needed, before they can be implemented in our scenario.

We only used one sensor (accelerometer) attached to the participants’ belt to detect the activity performed. This solution is unobtrusive and well suited for a long-term monitoring scenario, particularly in disabled or elder populations [34]. Using additional inertial sensors (eg, gyroscope or barometer) could improve the model performance, although at the cost of increased power requirements [35]. Similarly, the placement of the sensor on the body can affect the prediction accuracy for certain activities, as the optimal location is often a function of the activity to recognize [36]. Using multiple sensors on different body parts is also known to increase the accuracy [25], although it is likely to decrease patient compliance. Future studies should explore how these factors influence the accuracy of AR when patients use an assistive device.

### Conclusions

Guidelines on how to use wearable technology to track functional activities in populations other than young able-bodied are still lacking [37]. Our results suggest that AR models need to be validated on both the specific patient population and assistive device used and that personal models may confer an advantage only when trained on the specific assistive device used. Maximizing the reliability of AR models is a key enabling factor that will allow clinicians performing informed decisions based on the data. This is a necessary step to favor the deployment of such technology into the clinic.

## Abbreviations

AR: activity recognition
IQR: interquartile range
KAFO: knee-ankle-foot orthosis
OPUS-LEFS: orthotics prosthetics users survey for lower extremity functional status
